# Comparison between Hip Internal Rotation Assistive Device and Conventional Radiograph Positioning: An Experimental Study

**DOI:** 10.5704/MOJ.2211.002

**Published:** 2022-11

**Authors:** T Sirithiantong, T Sukhapanon, R Cheewatanakornkul

**Affiliations:** 1 Department of Orthopaedics, Hatyai Hospital, Hatyai, Thailand; 2 Department of Radiology, Hatyai Hospital, Hatyai, Thailand

**Keywords:** hip radiograph, internal rotation, internal rotation assistive device, radiograph positioning

## Abstract

**Introduction:**

Plain anteroposterior radiograph of the hip plays an important role in diagnosing hip pathology. However, one of the most common mistakes is image distortion because the hip is externally rotated due to natural femoral neck anteversion. Thus, the lower extremities should be internally rotated by 15°–20°. The researchers developed a hip internal rotation assistive device and compared it with conventional radiograph positioning.

**Materials and methods:**

A hip internal rotation assistive device was designed. This study consisted of 20 volunteers without clinical hip pathology. The volunteers were informed to do a hip radiograph twice to compare the efficacy of the developed device with a conventional positioning. The thickness of the lesser trochanter (TLT) was measured and interpreted by an orthopedist and a radiologist. Statistical significance and inter- and intra-observer reliabilities were analysed.

**Results:**

According to the orthopaedist’s measurement, the mean TLT distance was 4.42 + 3.2mm and 4.97 + 3.16mm for the conventional technique and assistive device, respectively, without statistical significance between both groups (*p* = 0.05). Consistent with the musculoskeletal radiologist, the mean TLT distance was 4.00 + 2.06mm for the conventional technique and 3.92 + 2.27mm for the assistive device, without statistical significance between both groups (*p* = 0.56). Intra-observer reliability was 0.900 and 0.898 for the orthopaedist and the radiologist, respectively. Interobserver reliability of the assistive device and conventional technique were 0.800 and 0.588, respectively.

**Conclusion:**

The efficacy of the developed device was similar to that of the conventional technique. Inter/intra-observer reliabilities were at a good agreement level in both methods. The developed device would also be useful in clinical applications, especially in decreasing unnecessary radiation exposure of medical personnel.

## Introduction

Plain radiographic examination is a basic and critical method for diagnosing hip disorders^1,2^. An anteroposterior (AP) hip radiograph that includes images of both sides of the hip provides information about treatment, especially in patients with hip fracture^3^. A plain hip radiograph is crucial for patients who need total hip arthroplasty^4,5^. The excessive hip external rotation film is related to choosing the inappropriate size of the prosthetic stem in pre-operative femoral templating^6^. Additionally, some hip pathology may be obscured by excessive hip external rotation, especially pathology at the femoral neck, such as femoral neck stress fracture, bone tumour, and others, in patients with hip pain^1^.

According to the anatomy of the femur with natural femoral neck anteversion, the AP view should be internally rotated by 15°–20° in positioning for a plain hip radiograph to accommodate the femoral neck anteversion^1,4,6-9^. However, the most common mistake is image distortion because the hip is externally rotated^1^. Unnecessary radiation exposure to medical personnel was a concern, which can contribute to cancer, such as leukaemia, thyroid cancer, breast cancer, radiation-induced lens opacities, and others, with radiation exposure overdosage^10-11^.

Therefore, the hip internal rotation assistive device was created to improve or replace conventional radiograph positioning. This device is a new tool that holds both lower limbs to maintain a hip position at 20° internal rotation. The efficacy of hip internal rotation between the developed assistive device was compared with the conventional radiograph positioning by medical personnel.

## Materials and Methods

**Fig. 1.**
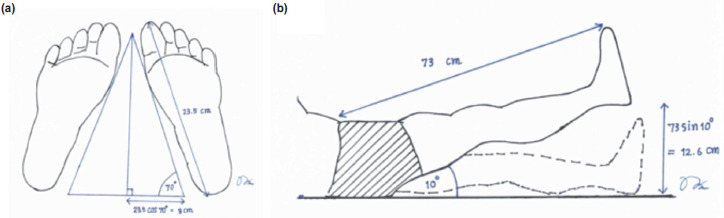
Trigonometry was used for calculating the size, length, and angles of this device. (a) Axial view and (b) Sagittal view.

The assistive device was designed based on average foot length (23.5cm), foot width (5.5cm), and true limb length (73cm) according to the approximation of anthropometric data of Thai people by the Ministry of Industry. The trigonometry was used to calculate the size, length, and angles of this device, and then the templating model was done as in Fig. 1.

**Fig. 2.**
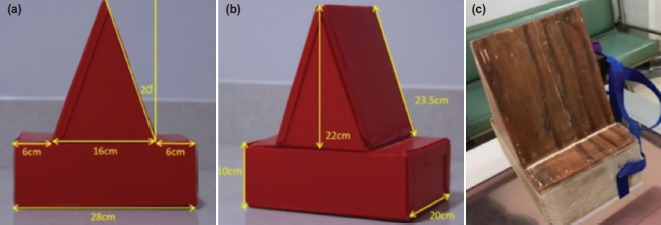
Hip internal rotation assistive device. Template design (a) axial view and (b) oblique view. (c) The final device was built with wooden material and a velcro strap.

The device was designed to elevate the lower limb from the base so that the lower limb can easily rotate. However, if hip flexion is >10°, it can decrease the accuracy of femoral neck-shaft angle measurement from a plain radiograph^7^. According to the average true limb length and hip flexion of 10°, we need to elevate the lower limb by <12.6cm as the trigonometry calculation requires. We design the device to elevate the lower limb by 10cm from the base. Finally, the triangular-like device is shown in Fig. 2.

**Fig. 3.**
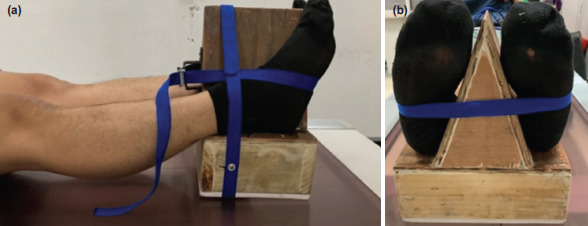
Radiograph positioning by Hip internal rotation assistive device. (a) Sagittal view and (b) axial view.

**Fig. 4.**
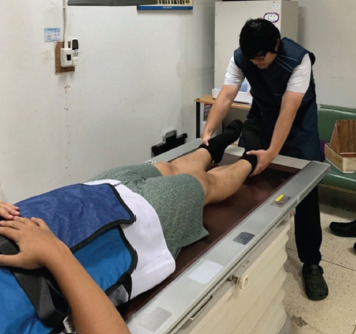
Conventional radiograph positioning by medical personnel.

The experimental study was performed under the Institutional Review Board approval from June to December 2020 (Research ethics committee of Hatyai hospital, protocol number 80/2562). For the inclusion criteria, 10 males and 10 females aged >18 years, without clinical hip abnormality by history taking and physical examination, were included in this study. The demographic data were recorded, including age, gender, body weight, height, body mass index (BMI), true leg length (measured from the anterior superior iliac spine to the medial malleolus), foot length (measured from the tip of the big toe to the most posterior part of the sole), sole width (measured from the distance between the medial and the lateral of the widest part of the hindfoot), and range of motion of internal–external hip rotation. All measurements were performed using a tape measure and goniometer by an orthopaedic surgeon (TeS). The subjects underwent hip AP radiographs two times using the assistive device (Fig. 3) and the conventional technique, respectively (Fig. 4). The lead apron was applied to reduce unnecessary radiation exposure to other body parts, especially the breast and the neck. The general radiograph machine [TOSHIBA MRAD-A80S with KONICA AERO DR 2 Detector Tokyo, Japan, 2015] used in this study had a radiation size of 80kVp and a current volume of 15-20mAs. The distance between the radiation receiver and the emitter head is 1.2m.

**Fig. 5.**
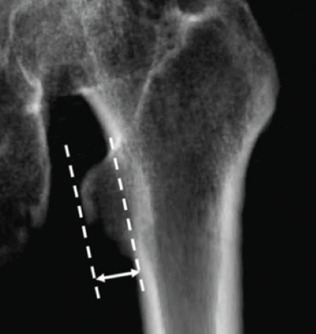
The thickness of the lesser trochanter is represented by the perpendicular distance between the two dotted lines, from the external prominent contour of the lesser trochanter to the femoral cortex3.

The radiographic data were collected by Picture Archiving and Communication system. Measurement and interpretation were measured two times, one month apart by the orthopaedist (ToS) and the musculoskeletal radiologist (RC). The thickness of the lesser trochanter (TLT) was measured and recorded by a blinded interpreter about the radiograph method. The adequate internal rotation was determined by TLT of <5mm^1,6^ on both sides of the hip (Fig. 5).

The demographic data were summarised in mean and range for age, body weight, height, BMI, leg length, foot length, sole width, and range of motion of internal-external rotation of the hip. Data were analysed using the Statistical Package for the Social Sciences software [IBM® SPSS® Statistics, United States of America] for statistical calculation. The TLT distance was recorded and compared to determine the statistically significant level using paired T-test. The quality of hip internal rotation was recorded and compared to determine statistical significance with the McNemar test between the conventional technique and the assistive device. Both orthopaedist and radiologist were required for reinterpretation after the first time for one month to analyse intra- and interobserver reliabilities. The reliability values were analysed using the Kappa statistics.

## Results

This study consisted of 20 volunteers (10 males and 10 females). The mean age of patients was 23 years (range: 2129), mean BMI was 23.12kg/m^2^ (range:18.29-34.55), mean leg length was 89.17cm (range: 74.5-105), mean body weight was 64.4kg (range: 46-85), mean height was 167.2cm (range: 150-181), mean hip internal rotation was 32.7° (range: 24-50), mean hip external rotation was 54.45° (range: 32-90), mean sole width was 5.59cm (range: 4.5-6.5), and mean foot length was 24cm (range: 21-27).

According to the TLT distance measured by the orthopaedist, the mean TLT distance was 4.42 + 3.2mm for the conventional radiographic positioning and 4.97 + 3.16mm for the hip internal rotation assistive device method, without statistical significance between both methods (*p* = 0.051). Consistent with the results of the musculoskeletal radiologist, the mean TLT distance was 4.00 + 2.06 mm for the conventional method and 3.92 + 2.27mm for the assistive device method, without statistical significance between both methods (*p* = 0.563).

The orthopaedist’s interpretation revealed a 60% and 55% adequate internal hip rotation in the conventional and assistive device methods, respectively, but without statistical significance (p = 0.651).

The musculoskeletal radiologist’s interpretation revealed a 65% and 60% adequate internal hip rotation in the conventional and assistive device method, respectively, but without statistical significance (*p* = 0.562).

The Kappa statistical analysis revealed that the orthopaedist and radiologist intra-observer reliabilities on the internal rotation assistive device were 0.900 and 0.898, respectively (almost perfect agreement) and interobserver reliability of the internal rotation assistive device and conventional radiographic positioning were 0.800 (substantial agreement) and 0.588 (moderate agreement), respectively.

## Discussion

The plain film radiograph plays an important role in diagnosing hip pathology^1^. The excessive external hip joint rotation affects the interpretation of hip radiographic images^3,9-11^. Some previous techniques suggested the use of hip radiographs, such as self-maintain alignment by patients^1^, Specific V^-^shaped positioning frame^2^, and foot map aid with weight-bearing film^12^, but all of them have no evidence or any details about efficacy. Therefore, the researchers initiated the idea of using a device to align the patient’s hip in internal rotation.

This research used the experimental study conducted to answer questions about the efficacy of the developed hip internal rotation assistive device. The strength of this research is the comparison and measurement in the same volunteer to reduce the anatomy variation that would affect the comparative study. Furthermore, the interpreters were blinded, thus unable to know how each radiograph image was performed. The TLT and the quality of hip internal rotation were not different for either the use of the developed hip internal rotation assistive device or the conventional radiographic positioning. Additionally, relatively good intra-and interobserver reliabilities were found.

However, either the device or the conventional radiograph positioning was used, the quality of hip internal rotation was similar, approximately 55%–65% of all AP hip radiographs. Additionally, the volunteers may not represent the general population. Some issues should be considered for research extension in designing a hip internal rotation assistive device to increase internal rotation, as well as the possible application to the outlier population, such as those with morbid obesity, etc.

In the future, we expect that the developed assistive device will reduce unnecessary radiation exposure in medical personnel. Better quality hip radiograph images will be obtained. These may be very beneficial when this device will be applied to patients with cognitive problems or the inability to adjust their position in clinical practice.

## Conclusion

From our experimental study, the efficacy of the developed hip internal rotation assistive device for internal hip rotation was identical to that of the conventional radiographic positioning by medical personnel with good inter and intra-observer reliabilities. The findings implied that the developed device would be useful in clinical applications, especially in decreasing unnecessary radiation exposure in medical personnel.
